# Plasma proteomic analysis reveals altered protein abundances in cardiovascular disease

**DOI:** 10.1186/s12967-018-1476-9

**Published:** 2018-04-17

**Authors:** Vasiliki Lygirou, Agnieszka Latosinska, Manousos Makridakis, William Mullen, Christian Delles, Joost P. Schanstra, Jerome Zoidakis, Burkert Pieske, Harald Mischak, Antonia Vlahou

**Affiliations:** 1 Biomedical Research Foundation, Academy of Athens, 4 Soranou Ephessiou Street, 115 27 Athens, Greece; 2 Mosaiques Diagnostics GmbH, Rotenburger Straße 20, 30659 Hannover, Germany; 3 Institute of Cardiovascular and Medical Sciences, University of Glasgow, 126 University Place, Glasgow, G12 8TA UK; 4 Institut National de la Santé et de la Recherche Médicale (INSERM), U1048, Institute of Cardiovascular and Metabolic Disease, Toulouse, France; 5 Université Toulouse III Paul-Sabatier, Toulouse, France; 6 Deutsches Herzzentrum Berlin, Augustenburger Pl. 1, 13353 Berlin, Germany

**Keywords:** Cardiovascular disease, Proteomics, Plasma, LC–MS/MS, Proteomic dataset, Biomarker, Drug discovery

## Abstract

**Background:**

Cardiovascular disease (CVD) describes the pathological conditions of the heart and blood vessels. Despite the large number of studies on CVD and its etiology, its key modulators remain largely unknown. To this end, we performed a comprehensive proteomic analysis of blood plasma, with the scope to identify disease-associated changes after placing them in the context of existing knowledge, and generate a well characterized dataset for further use in CVD multi-omics integrative analysis.

**Methods:**

LC–MS/MS was employed to analyze plasma from 32 subjects (19 cases of various CVD phenotypes and 13 controls) in two steps: discovery (13 cases and 8 controls) and test (6 cases and 5 controls) set analysis. Following label-free quantification, the detected proteins were correlated to existing plasma proteomics datasets (plasma proteome database; PPD) and functionally annotated (Cytoscape, Ingenuity Pathway Analysis). Differential expression was defined based on identification confidence (≥ 2 peptides per protein), statistical significance (Mann–Whitney *p* value ≤ 0.05) and a minimum of twofold change.

**Results:**

Peptides detected in at least 50% of samples per group were considered, resulting in a total of 3796 identified proteins (838 proteins based on ≥ 2 peptides). Pathway annotation confirmed the functional relevance of the findings (representation of complement cascade, fibrin clot formation, platelet degranulation, etc.). Correlation of the relative abundance of the proteins identified in the discovery set with their reported concentrations in the PPD was significant, confirming the validity of the quantification method. The discovery set analysis revealed 100 differentially expressed proteins between cases and controls, 39 of which were verified (≥ twofold change) in the test set. These included proteins already studied in the context of CVD (such as apolipoprotein B, alpha-2-macroglobulin), as well as novel findings (such as low density lipoprotein receptor related protein 2 [LRP2], protein SZT2) for which a mechanism of action is suggested.

**Conclusions:**

This proteomic study provides a comprehensive dataset to be used for integrative and functional studies in the field. The observed protein changes reflect known CVD-related processes (e.g. lipid uptake, inflammation) but also novel hypotheses for further investigation including a potential pleiotropic role of LPR2 but also links of SZT2 to CVD.

**Electronic supplementary material:**

The online version of this article (10.1186/s12967-018-1476-9) contains supplementary material, which is available to authorized users.

## Background

Cardiovascular disease (CVD) is the leading cause of morbidity and mortality worldwide [1]. CVD comprises multiple clinical conditions affecting the heart and blood vessels which may vary in severity. These include coronary artery disease, peripheral arterial disease, rheumatic heart disease, heart failure, ischemic stroke, myocardial infarction, cardiomyopathy, and others [1]. Common molecular features that have been associated to CVD include oxidative stress, inflammation and extracellular matrix remodeling [2, 3]. However, understanding the molecular mechanisms underlining the onset and progression of the CVD remains incomplete, hindering the development of methods for prevention, early diagnosis, prognosis and targeted therapies. Early stages of CVD can be, at least in part, reversible, but since they are clinically asymptomatic, their diagnosis is challenging. Furthermore, the available treatment strategies mainly aim to suppress the symptoms (i.e. hypertension, hypercholesterolemia and heart load) and not the etiologies of the disease [4, 5]. Thus, a need for deeper understanding of the key molecular mechanisms responsible for the observed pathology in CVD clearly still exists.

A consensus that has arisen the last years is that to tackle a multifactorial and complex disease, such as CVD, a global view of the underlying molecular pathophysiology is required [6]. Towards this direction, several genome-wide association studies (GWASs) have been conducted in the recent years that support the existence of a genetic impact even in late-onset CVD. Smith et al. reviewed statistically robust (with regard to multiple testing, p < 5 × 10^−8^) GWAS on late-onset CVD and relative traits. The analysis highlighted 92 genetic loci being associated with coronary artery disease, carotid artery disease, ischemic stroke, aortic aneurysm, peripheral vascular disease, atrial fibrillation, valvular disease and correlates of vascular and myocardial function. While only a few of these variations have an established underlying mechanism in the disease, the novel molecular players and pathways revealed by these studies provide new insights in the pathophysiology of the disease [7]. However, the biggest part of heritability of cardiovascular traits is not yet explained. This could be due to the low minor allele frequencies and rare variants that are excluded in GWASs, as well as the gene-environment and gene–gene (epistatic) interactions that are usually not taken into account [8]. Along the same lines, multiple studies on gene expression in CVD have been conducted with the employment of transcriptomics approaches. Chen et al. compared 15 studies that yielded in total 706 genes differentially expressed between coronary artery disease and controls, but only 23 of these genes were replicated in 2, maximal 3 different studies [9]. These differentially expressed genes included annexin A3, fibrinogen alpha chain, guanylate kinase 1, haptoglobin and interferon gamma. The low number of reproducible results may be due to small study cohorts and genetic variability [9].

In parallel to the studies on genetic polymorphisms and transcriptome, several studies investigating changes at the protein level in tissue, plaques, plasma and urine associated with CVD have been conducted (reviewed in [10]). Investigation at the proteome level is of special value when studying disease associated mechanisms, as proteins integrate genomic information with environmental impact, thus changes at the protein level are expected to reflect the disease phenotype especially well. Based on the non- (urine) or minimally invasive (plasma) means for their collection, analysis of the urine and plasma proteome in association to CVD has gained special attention. Urinary protein markers that have been suggested in this context include orosomucoid 1 for chronic heart failure [11]; CD14 in stable coronary artery disease [12]; and uromodulin in hypertension [13]. Extensive analysis of the urine peptidome, using capillary electrophoresis–mass spectrometry (CE–MS), has also been described revealing biomarker profiles for coronary artery disease [14]; heart failure [15]; stroke [16]; hypertension with left ventricular diastolic dysfunction [17, 18]; and acute coronary syndromes [19].

Multiple protein changes have been also reported to date in plasma. These studies, involving use of both gel-based and non-gel-based techniques (reviewed in [20]), addressed the identification of features associated with specific CVD phenotypes, including abdominal aneurism progression [21]; fibrin clot-bound proteins in acute myocardial infarction [22]; stable coronary artery disease pathophysiology [23]; prognostic biomarkers in peripheral arterial occlusive disease [24]; venous thromboembolism risk prediction [25]; and extracellular matrix remodeling in hypertrophic cardiomyopathy [26]. Significant changes in multiple proteins included various forms of apoliproteins [23]; thrombospondin 1 [10]; alpha 2 macroglobulin [10]; transthyretin [24]; complement factor B [24]; platelet-derived growth factor β [25]; and fibronectin [26].

**Fig. 1:**
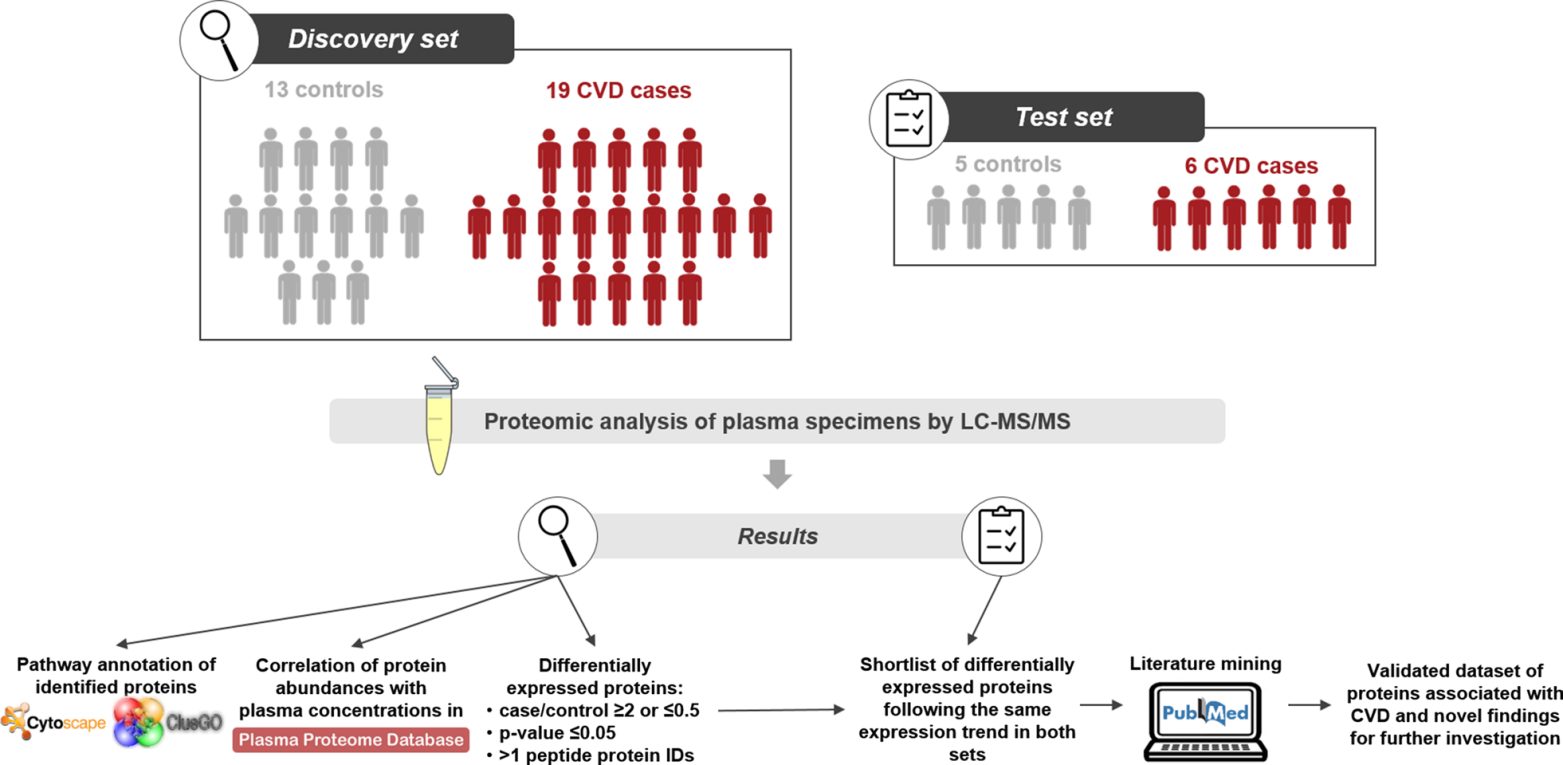
Summary of the workflow followed in the present study. For increased validity, subjects were divided randomly into discovery and test sets. After proteomic analysis of the plasma specimens, pathway annotation was performed for the discovery set proteins and their abundance was correlated with the respective plasma concentrations in the plasma proteome database. Differentially expressed proteins were defined with the following criteria: case/control ratio ≥ 2 or ≤ 0.5; Mann–Whitney p-value ≤ 0.05; protein identifications based on multiple peptides. The differentially expressed proteins with identical expression trend in both sets were shortlisted and extensive literature mining was performed. The resulting validated dataset consists of proteins previously found associated with CVD and novel findings to be further investigated in the context of CVD pathophysiology

Complementing existing studies and to further improve on global understanding of molecular mechanisms of CVD, we investigated plasma proteomic changes common to different clinical etiologies and severity of CVD from patients undergoing vascular surgery or organ donors, where vascular tissue was available for assessment of CVD. The comparison is made to controls of expected high inflammatory profile (donors deceased from accidents) without any cardiovascular symptom/event, and results are analyzed in the context of existing relevant literature (workflow is presented in Fig. 1). Multiple significant findings include changes previously reported at the GWAS and other molecular levels, collectively providing a well characterized and comprehensive protein dataset to support further data integration studies in the field.

## Methods

### Clinical samples

Blood plasma specimens were collected within the context of a prospective study on CVD (part of the FP7 project SysVASC: Systems Biology to Identify Molecular Targets for Vascular Disease Treatment), from control participants with no cardiovascular background (n = 13) and CVD patients (n = 19) in Medical University of Graz, Graz, Austria. All control and over 40% of the CVD (n = 8) samples were collected from deceased donors. The study was approved by the respective local ethics committee (approval number: 26-355 ex 13/14). CVD subjects were characterized by established vascular disease and had at least one of the following conditions: peripheral artery disease; coronary artery disease; arterial hypertension; cerebral hemorrhage; or carotid stenosis. The characteristics of the participants in this study are presented in Table 1.

**Table 1:** Characteristics of the participants

Variable	Control group (*n* = *13*)	Case group (*n* = *19*)
Demographics		
Gender [n (%)]	12 (92.3%) M, 1 (7.7%) F	10 (52.6%) M, 9 (47.4%) F
Age (years)	42.50 ± 16.8	65.8 ± 10.4
Physical characteristics		
Weight (kg)	82.5 ± 8.7	75.1 ± 10.6
Height (cm)	177.8 ± 9.0	169.3 ± 7.3
Body mass index (kg/m^2^)	26.1 ± 2.1	26.2 ± 3.3
Clinical information [n (%)]		
Hypertension	2 (15.4%)	18 (94.7%)
Obesity	1 (7.7%)	2 (10.5%)
Diabetes	0 (0%)	5 (26.3%)
Dyslipoproteinemia	0 (0%), 1 (7.7%) NA	2 (10.5%)
Left ventricular hypertrophy	1 (7.7%)	6 (31.6%)
Angina pectoris	0 (0%)	1 (5.3%)
Intermittent claudication	0 (0%), 1 (7.7%) NA	7 (36.8%)
Myocardial infarction	0 (0%)	3 (15.8%)

For gender: *M* male, *F* female. Hypertension occurrence corresponds to systolic blood pressure ≥ 140/90 mmHg. Obesity is defined by a body mass index > 30 kg/m^2^. For dyslipoproteinemia all the following measurements have to occur: total cholesterol ≥ 5.2 mmol/l (≥ 200 mg/dl); low-density lipoprotein (LDL) cholesterol ≥ 3.4 mmol/l (≥ 130 mg/dl); high-density lipoproteins (HDL) cholesterol ≤ 1.0 mmol/l (≤ 40 mg/dl); and triglycerides ≥ 1.7 mmol/l (≥ 150 mg/dl)

*NA* not available data

### Sample preparation

Plasma samples were processed with the filter-aided sample preparation protocol, as previously described by Wisniewski et al. [27]. Specifically, protein concentration of plasma was determined by Bradford assay and 200 µg of protein per sample was mixed with 0.2 ml of 8 M urea in 0.1 M Tris/HCl, pH 8.5 (urea buffer). Reduction was performed with 0.1 M dithioerythritol (DTE) for 20 min (room temperature—RT) and the samples were loaded into 30 kDa Amicon Ultra Centrifugal Filters, Ultracel (Merck Millipore). The filters were then centrifuged at 16,000*g* for 15 min and washed twice with 0.2 ml of urea buffer. The concentrates were then mixed with 0.1 ml of 50 mM iodoacetamide in urea solution and incubated in the dark, at RT for 20 min (alkylation), followed by centrifugation for 10 min. Then, the filters were washed once with 0.1 ml of urea solution and twice with 0.1 ml of 50 mM NH_4_HCO_3_, pH 8.5. Finally, samples were digested with trypsin (proteomics grade) overnight, in the dark at RT (trypsin/protein ratio: 1/100) and the peptides were collected by centrifugation of the filter units for 10 min at 16,000*g* in clean tubes. The filters were washed once more with 40 µl of 50 mM NH_4_HCO_3_ and eluted peptides were collected in the same tube. The eluates were lyophilized and stored at − 20 °C until further processing.

### LC–MS/MS analysis

Five microgram of protein digest was loaded onto a Dionex Ultimate 3000 RSLS nano flow system (Dionex, Camberly UK). After loading onto a Dionex 0.1 × 20 mm 5 μm C18 nano trap column at a flow rate of 5 μl/min in 0.1% formic acid and 2% acetonitrile, samples were applied onto an Acclaim PepMap C18 nano column 75 μm × 50 cm (Dionex, Sunnyvale, CA, USA), 2 μm 100 Å at a flow rate of 0.3 μl/min. The trap and nano flow column were maintained at 35 °C. The samples were eluted with a gradient of solvent A: 0.1% formic acid and 2% acetonitrile versus solvent B: 0.1% formic acid and 80% acetonitrile starting at 1% B for 5 min rising to 5% B at 10 min then to 25% B at 360 min and 65% B at 480 min. The column was then washed and re-equilibrated prior to injection of the next sample. The eluent was ionized using a Proxeon nano spray ESI source operating in positive ion mode into an Orbitrap Velos FTMS (Thermo Finnigan, Bremen, Germany). Ionization voltage was 2.6 kV and the capillary temperature was 275 °C. The mass-spectrometer was operated in MS/MS mode scanning from 380 to 1600 amu. The resolution of ions in MS1 was 60,000 and 7500 for higher-energy collisional dissociation (HCD) MS2. The top 20 multiply charged ions were selected from each scan for MS/MS analysis using HCD at 40% collision energy. AGC settings were 1,000,000 for full scan in the FTMS and 200,000 for MSn. Dynamic exclusion was enabled with a repeat count of 1, exclusion duration of 30 s.

### MS data processing

Protein identification was performed with Proteome Discoverer 1.4 (Thermo Scientific) using the SEQUEST search engine [28]. Protein search was performed against the SwissProt human protein database [29] (30.05.2016) containing 20,197 reviewed entries. The following search parameters were applied: (i) precursor mass tolerance: 10 ppm and fragment mass tolerance: 0.05 Da; (ii) full tryptic digestion; (iii) max missed cleavage sites: 2; (iv) static modifications: carbamidomethylation of cysteine; (v) dynamic modifications: oxidation of methionine; (vi) event detector mass precision: 2 ppm; (vii) precursor mass: 600–5000 Da; (viii) collision energy: 0–1000 eV; (ix) target FDR (strict): 0.01; (x) target FDR (relaxed): 0.05; (xi) FDR validation based on: q-value. Obtained results were further processed by applying the following filters: (i) high, medium and low confidence peptides (FDR < 10%); (ii) peptide rank up to 5; (iii) peptide grouping was enabled and protein grouping was disabled. The list of peptides was exported and processed using a clustering approach that allows combining the data sets acquired in the course of multiple MS runs. This approach groups features (as defined by calibrated retention time and mass) into clusters, followed by the sequence assignment. In this way, data are harmonized, and consistency in sequence assignment when multiple samples are analyzed in individual experiments is achieved. In brief, data were processed in the following steps: (1) calibration: to adjust for sample to sample LC retention time (rt) shifts, rt was calibrated using LOWESS (locally weighted scatterplot smoothing) non-parametric regression method [30]. To identify and calibrate respective peptides across the whole study, a peptide list from one sample (i.e. sample 27—control) covering the full mass and rt range was selected as reference, based on which alignment/calibration of rt of all samples was performed. (2) Clustering: peptides, as defined by mass and calibrated rt, from all exports from Proteome Discoverer were compiled into a single file. This compiled list was used as an input file for clustering. Clustering was then performed according to the following algorithm: all data points (pairs of mass and rt) were placed in a 2-dimensional plane. Clusters (mass window of ± 5 ppm and retention time window of ± 5% of the feature’s rt) were defined and moved until capturing most data points. The center of these clusters (pairs of mass and rt) were compiled in a “cluster list”, with each of the clusters assigned with a unique identifier. Only clusters with at least 2 members were considered for further analysis. (3) Matching and sequence assignment: as described above, at this stage each cluster includes a set of features (peptides) within a certain “window” of mass and rt. For each cluster, the sequence with the highest frequency across the study was selected as representative. In case of a tie, the sequence with the highest Xcorr was selected. (4) Protein annotation: for each assigned sequence, Uniprot ID was annotated with protein name and gene symbol, based on the respective Uniprot database. In the case of one peptide being assigned to multiple proteins, the protein represented with highest frequency across the cluster list was selected. (5) Retrieve peptide information for further quantification: peptide area, confidence, Xcorr and mass/retention time information were retrieved from the peptide lists of each sample according to their cluster IDs. Only peptides < 5 ppm difference between experimental and theoretical mass were considered. (6) Quantification: Samples were randomly divided into discovery and validation set based on the rule 2/3 (n = 22, 13 cases and 8 controls) and 1/3 (n = 11, 6 cases and 5 controls), respectively. Each set of samples was analyzed separately as follows: for a limited number of sequences for which no peptide area could be retrieved by proteome discoverer (this is a well-known, but not yet corrected problem of this software), the missing values were replaced by the mean area values of that group. When the peptide was not identified in the particular sample, the missing values were replaced with zero. For the discovery set, only peptides reported in more than 50% of the samples of at least one group were included in the differential expression analysis, while no filters were applied in the test set analysis. Subsequently, part per million (ppm)-normalization of the peptide peak areas was conducted according to the following formula: normalized peak area = (peptide peak area/total peak area) × 10^6^. Protein abundance in each sample was calculated as the sum of all normalized peptide areas for a given protein, as described previously [31]. Statistical analysis was based on the Mann–Whitney test performed using R package. Proteins with p-value ≤ 0.05 were considered as statistically significant.

### Functional analysis

Functional analysis was performed with the ClueGO plug-in [32] in Cytoscape 3.4.0 [33] as well as the Ingenuity Pathway Analysis software (QIAGEN Inc.) [34]. In regard to the ClueGO analysis, ontologies were retrieved from REACTOME pathways database (updated on September 4, 2017) and only statistical significant pathways (Bonferroni corrected p-value ≤ 0.05, two-sided hypergeometric test) were taken into account. For the remaining parameters, default settings were used. Results were simplified based on biological relevance and only the leading term from each group is presented.

### Correlation analysis

Spearman rank correlation analysis was performed using IBM SPSS Statistics for Windows, Version 22.0 after logarithmic transformation of the values.

## Results

### Discovery set analysis: establishing validity of the proteomics dataset

In total, 32 plasma samples, 13 controls and 19 CVD cases, were analyzed by high-resolution LC–MS/MS. To establish confidence in the validity of the results from the differential expression analysis, the samples were randomly divided into discovery (2/3 of the samples; corresponding to 21 samples—13 cases and 8 controls) and test (1/3 of the samples; corresponding to 11 samples—6 cases and 5 controls) sets. In the discovery set, only peptides detected in at least 50% of samples per group (cases or controls) were considered, resulting in a total of 3796 identified proteins. Of these, 838 protein identifications were based on at least 2 peptides. The full list of identified peptides and proteins is provided in Additional file 1. To confirm the biological relevance of this dataset, pathway annotation was performed using the ClueGO plug-in of Cytoscape platform (Additional file 2). As shown, the detected proteins segregated into (plasma) biologically relevant pathways such as complement cascade, scavenging heme from plasma, fibrin clot formation and platelet degranulation (these being among the statistically significant formed pathways—Additional file 2). When only identifications based on at least 2 peptides were included in the analysis, HDL assembly and TGF-b signaling also emerged among the statistically significant pathways (Additional file 2). Collectively, this phenotyping of the findings via pathway annotation verified that the proteins identified via the applied proteomics approach are biologically relevant and the ones expected to be found in plasma based on the current knowledge.

**Fig. 2:**
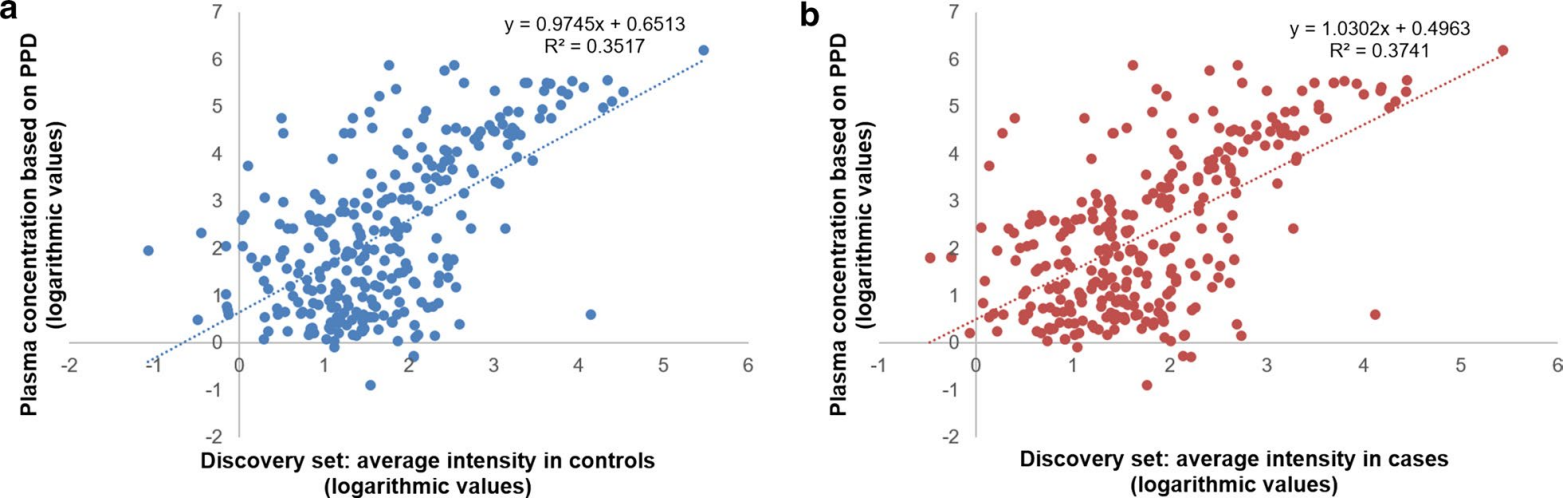
Scatter plots showing the relative abundances of identified proteins and their plasma concentration in PPD. **a** Proteins identified in controls and **b** proteins identified in cases. Scatter plots were created after logarithmic transformation of the values

To assess the reliability of the applied quantification approach, the relative abundance of proteins from the discovery set was compared to the respective absolute concentrations reported in the plasma proteome database (PPD) (http://plasmaproteomedatabase.org/index.html) [35]. For this comparison, PPD entries originating from spectral counting experiments on plasma were considered. This corresponded to 299 proteins (286 proteins in the controls, 288 proteins in the cases) reported in both PPD and our study (listed in Additional file 3). Spearman rank correlation analysis revealed significant correlation between the reported PPD concentrations and the observed protein abundances in our datasets (r_s_ = 0.521, p-value < 0.0001 for controls; and r_s_ = 0.536, p-value < 0.0001 for cases). Scatter plots of these comparisons are presented in Fig. 2. This result supports that the applied quantification strategy is appropriate incorporating state of the art knowledge.

### Test set analysis: differential expression analysis reveals expected and novel protein changes

To increase reliability in the differential expression analysis in the discovery set, given also the relatively small sample size, only proteins identified based on at least 2 peptides were considered, and differential expression was defined based on statistical significance (Mann–Whitney p-value ≤ 0.05) and change by at least twofold in the cases versus controls. A total of 100 proteins were found to meet these criteria (listed in Additional file 1). These findings were subsequently examined for validity in an independent test set of 11 samples, including 5 controls and 6 cases (the full list of peptides and proteins identified in the test set is provided in Additional file 3). Spearman rank correlation analysis revealed significant correlation between the protein abundances of discovery and test sets (r_s_ = 0.850, p-value < 0.0001 for controls; and r_s_ = 0.893, p-value < 0.0001 for cases). Out of the 100 differentially expressed proteins, 39 proteins were detected at the same expression trend (up- or down-regulated) and with a fold change of at least 2, when comparing cases and controls in the test set (Table 2, Additional file 3). Of these 39 proteins, 9 showed statistically significant changes (Mann–Whitney p-value ≤ 0.05) also in the test set (Table 2, Additional file 3) and thirteen were described in the past as associated with cardiovascular disease, further supporting the validity of our approach. As shown in Table 2, the latter proteins included alpha-2-macroglobulin; apolipoprotein B; heparin cofactor 2; lipopolysaccharide-binding protein; latent-transforming growth factor beta-binding protein 2; and others.

**Table 2:** Shortlisted differentially expressed proteins following the same expression trend in both discovery and test sets

Uniprot ID	Name	Discovery set			Test set			CVD relevant publications
		Number of peptides	Cases/controls	Mann–Whitney p-value	Number of peptides	Cases/controls	Mann–Whitney p-value	
Q96M60	Protein FAM227B	2	0.44	*7.58E−04*	2	0.38	*8.11E−03*	
Q03164	Histone-lysine *N*-methyltransferase 2A	2	0.38	*9.84E−04*	2	0.12	*8.11E−03*	
Q9NVW2	E3 ubiquitin-protein ligase RLIM	3	2.66	*4.23E−03*	4	2.46	*8.11E−03*	
O60494	Cubilin	2	15.19	*1.18E−03*	2	27.19	*1.29E−02*	
P01023	Alpha-2-macroglobulin	64	2.37	*1.27E−03*	82	2.50	*1.37E−02*	[39, 40]
Q02880	DNA topoisomerase 2-beta	3	3.86	*2.98E−03*	9	3.25	*1.37E−02*	
Q8WWI1	LIM domain only protein 7	2	3.26	*4.23E−03*	2	3.33	*2.25E−02*	[76]
O75665	Oral-facial-digital syndrome 1 protein	2	3.01	*1.86E−02*	2	3.36	*2.25E−02*	[77]
P36957	Dihydrolipoyllysine-residue succinyltransferase component of 2-oxoglutarate dehydrogenase complex, mitochondrial	2	2.67	*4.61E−02*	2	5.50	*3.53E−02*	[78]
O75445	Usherin	2	2.50	*3.35E−03*	5	9.25	5.52E−02	
P05546	Heparin cofactor 2	8	2.99	*3.36E−03*	17	3.99	5.52E−02	[37, 79]
Q8IZU0	Protein FAM9B	2	2.36	*3.36E−03*	2	4.14	5.52E−02	
A0AUZ9	KAT8 regulatory NSL complex subunit 1-like protein	2	3.30	*1.53E−02*	2	2.11	5.52E−02	
Q92766	Ras-responsive element-binding protein 1	2	2.31	*3.88E−02*	2	3.35	6.73E−02	
Q5TEZ5	Uncharacterized protein C6orf163	4	2.13	*1.25E−02*	4	2.28	8.28E−02	
P54317	Pancreatic lipase-related protein 2	2	0.01	*4.51E−03*	3	Only in controls	9.96E−02	
Q9H6S0	Probable ATP-dependent RNA helicase YTHDC2	3	5.23	*4.60E−03*	6	2.31	1.21E−01	
P01266	Thyroglobulin	3	0.33	*9.90E−03*	4	0.46	1.21E−01	
P08185	Corticosteroid-binding globulin	2	2.65	*3.26E−02*	4	3.35	1.21E−01	[80]
Q5T200	Zinc finger CCCH domain-containing protein 13	3	8.07	*4.18E−03*	3	5.71	1.35E−01	
P18428	Lipopolysaccharide-binding protein	5	0.29	*7.55E−04*	8	0.43	1.71E−01	[81]
P98164	Low-density lipoprotein receptor-related protein 2	5	4.00	*1.25E−02*	10	3.92	1.71E−01	[58]
Q5T011	Protein SZT2	2	0.33	*1.86E−02*	3	0.37	1.71E−01	
P04114	Apolipoprotein B-100	68	2.47	*3.26E−02*	135	2.16	1.71E−01	[41, 42, 46]
Q9NS86	LanC-like protein 2	3	2.54	*4.64E−02*	3	2.76	1.71E−01	
Q9H7L9	Sin3 histone deacetylase corepressor complex component SDS3	2	4.70	*1.84E−02*	4	2.50	2.00E−01	
O60884	DnaJ homolog subfamily A member 2	2	6.36	*2.67E−03*	2	2.21	2.31E−01	
Q5T481	RNA-binding protein 20	4	4.84	*9.84E−04*	4	4.61	2.35E−01	[47, 82]
Q5VZL5	Zinc finger MYM-type protein 4	2	0.36	*5.30E−03*	2	0.38	2.62E−01	
O15553	Pyrin	2	11.75	*1.72E−03*	2	2.65	2.74E−01	[49–51, 83]
Q96PV0	Ras/Rap GTPase-activating protein SynGAP	2	0.15	*7.33E−03*	3	0.49	3.11E−01	
Q8N436	Inactive carboxypeptidase-like protein X2	2	0.46	*1.84E−02*	3	0.37	3.11E−01	
Q14767	Latent-transforming growth factor beta-binding protein 2	4	0.28	*7.58E−04*	6	0.43	3.15E−01	[84]
Q8IWB6	Inactive serine/threonine–protein kinase TEX14	2	4.64	*3.84E−02*	4	2.43	3.60E−01	
Q5T9S5	Coiled-coil domain-containing protein 18	2	0.37	*2.01E−02*	3	0.25	3.89E−01	
Q8N163	Cell cycle and apoptosis regulator protein 2	2	0.21	*3.51E−02*	3	0.17	3.89E−01	
Q9GZU1	Mucolipin-1	2	0.35	*1.46E−02*	3	0.48	4.10E−01	
O43301	Heat shock 70 kDa protein 12A	2	3.16	*1.68E−02*	2	3.91	4.11E−01	[85]
A6NES4	Maestro heat-like repeat-containing protein family member 2A	2	0.28	*7.55E−04*	3	0.24	6.48E−01	

Mann–Whitney p-values in italics font indicate values ≤ 0.05

Interestingly, molecular pathways that these 39 proteins participate in, based on the Ingenuity Pathway Analysis (IPA), included acute phase signaling; LXR/RXR activation; interleukin-6 signaling; coagulation system; and iNOS signaling (Additional file 4; canonical pathways), suggesting potential impact on these processes based on the observed protein abundance changes in CVD. In fact, and despite the small input dataset (39 proteins), most of the aforementioned pathways (with the exception of iNOS signaling) were predicted to be changing at statistically significant levels in the CVD cases versus controls (Additional file 4).

Along the same lines, IPA predicted significant enrichment of various cardiovascular system- and lipid metabolism-related processes based on the 39 verified proteins; these included uptake of lipid; activation of vascular endothelial cells; atherogenesis; hypercholesterolemia; vasculogenesis; dilated cardiomyopathy; all emerging as statistically significant functions reflected in the verified proteins (Additional file 4; biological functions).

## Discussion

The proteomic findings presented in this study provide a good source of information for further analysis and systems biology approaches. Proteomic data were generated from well characterized clinical specimens following a high resolution proteomic approach. The latter has been extensively optimized to provide a comprehensive view of the proteome, without performing abundant plasma protein depletion, frequently associated with technical variability and loss of information (from non-specific protein removal due to antibody cross-reactivity or protein binding) [36]. The identified proteins were investigated regarding their biological relevance, by pathway annotation, and, regarding the reliability of the quantification approach, by cross-correlation with the respective PPD absolute concentrations. As expected, biological pathways, such as acute phase response, interleukin-6, iNOS, and atherosclerosis signaling, and functions, such as activation of vascular endothelial cells, atherogenesis, hypercholesterolemia and transport of lipid, were predicted to be deregulated in CVD based on the observed protein changes.

To our knowledge, this is the first untargeted proteomic study on patients with various CVD-related conditions and severities. Thus, this study is expected to represent a valid foundation and initiation point for further investigation of common molecular mechanisms that underline the variety of CVD traits and conditions.

Interestingly, 13 out of the 39 verified proteins found in our analysis have been already associated with CVD in the literature (Table 2). As examples, Li et al. recently reported that plasma levels of alpha-2-macroglobulin positively correlated with higher vulnerability of carotid plaques, and increased risk of stroke [37]. In a proteomic study of plasma micro-particles, alpha-2-macroglobulin was also found upregulated in patients after deep venous thrombosis in comparison to healthy subjects [38]. In addition, higher alpha-2-macroglobulin serum levels were indicators of cardiac complications in HIV patients [39] and myocardial infarction in diabetic patients [40].

Along the same lines, apolipoprotein B, which we found upregulated in CVD, is widely considered as a major causal agent of atherosclerosis, is a structural component of plasma lipoproteins [i.e. chylomicron remnants, very low-density lipoprotein (VLDL), intermediate-density lipoprotein, LDL, and lipoprotein(a)] and mediates cholesterol transport and removal in vascular wall [41]. Apoliprotein B-100, the form identified in our study, plays a key role in the binding of LDL particles (prominent driver of atherogenesis) to the LDL receptor, allowing cells to internalize LDL and thus to absorb cholesterol [42]. Plasma levels of apoliprotein B and ratio of apoliprotein B/apoliporotein A–I (controls: 0.21, cases: 0.29) are increased in CVD compared to controls in our analysis. This finding is in agreement with studies correlating increased plasma apolipoprotein B and apoliprotein B/apoliporotein A–I ratio with myocardial infarction [43–45] and ischemic stroke [46].

In addition, mutations and polymorphisms in genes that code for some of the validated differentially expressed proteins detected in the present analysis have been connected with CVD. This suggests that these proteins may play important roles in the pathogenesis of CVD, and/or might also serve as early disease markers, hence are worth further investigation. Examples include: Rbm20 (encoding for RNA-binding protein 20) whose mutations in exon 9 of the gene have been linked to familial dilated cardiomyopathy and associated with young age at diagnosis, end-stage heart failure, and high mortality [47, 48]; and MEFV (pyrin encoding gene) whose mutations are a risk factor for early coronary artery disease [49] and are associated with childhood polyarteritis nodosa [50] and vascular complications in Behçet’s disease [51].

Within the verified proteins of our study and with no previous report in CVD to the best of our knowledge, low-density lipoprotein receptor-related protein 2 (LRP2) was also detected at increased levels in the plasma of CVD subjects in comparison to controls. With LRP2 interacting with both apolipoprotein B-100, mediating endocytosis of low density lipoproteins [52], and cubilin, (also upregulated in CVD plasma compared to controls), facilitating endocytosis of high density lipoproteins [53], we may hypothesize a central role of this protein in the accumulation of lipoproteins and the abnormal cholesterol metabolism occurring during CVD [54].

The central role of LRP2 in CVD is further supported via its function as an auxiliary receptor that controls sonic hedgehog (Shh) signaling [55]. Both knock-out mice for Shh [56] and mice carrying mutations in Lrp2 [57] exhibit cardiac outflow tract septation defects. Moreover, Baardman et al. showed that Lrp2 knock-out mice carry a variety of severe cardiovascular abnormalities, such as aortic arch anomalies, ventricular septal defects, overriding of the tricuspid valve and marked thinning of the ventricular myocardium [58]. Collectively, and considering the observed cardioprotective and angiogenic effects of Shh signaling in adulthood [59–61], further investigation of LRP2 as a potential central and pleiotropic node in CVD is suggested.

One additional finding with no previous association to CVD, is Ras-responsive element-binding protein 1 (RREB1), found at increased levels in the plasma of CVD patients compared to controls. Interestingly, RREB1 is an upstream regulator of the renin-angiotensin system acting as a transcriptional suppressor of the angiotensinogen gene [62, 63]. Since angiotensinogen is the precursor molecule of angiotensins I and II that induce vasoconstriction and blood pressure increase [64, 65], we may hypothesize that RREB1 may reflect the upregulation of a compensatory mechanism targeting to reduce hypertension.

Protein SZT2, that was found downregulated in CVD patients’ plasma, has been mainly associated with epileptogenesis and human brain development [66]. Recent reports support that SZT2 deficiency leads to increased mechanistic target of rapamycin complex 1 (mTORC1) signaling [67, 68]. Additionally, hypertrophic signals (such as pressure overload, β-adrenergic stimulation and angiotensin II) can also induce mTORC1 signaling in the heart, resulting in significant phenotypic impact: mTORC1 can induce cardiac hypertrophy as a compensatory mechanism to maintain function during pressure overload but also, and depending on the molecular background, can induce pathological cardiac hypertrophy [69]. Therefore, collectively, we may propose that the observed SZT2 decrease in CVD may be linked to increased mTORC1 signaling, a hypothesis meriting further investigation.

Another protein with no previous association to CVD included in the verified list, is lanC-like protein 2 (LANCL2) found to be at increased plasma levels in CVD patients. LANCL2 is the molecular target of abscisic acid, a natural phytohormone and an endogenous hormone with immune modulatory function [70]. Magnone et al. reported that abscisic acid participates in the development of atherosclerosis through the activation of monocytes and vascular smooth muscle cell responses, and its levels in atherosclerotic plaques are significantly higher than in normal vessels [71]. On the other hand, Guri et al. showed that feeding apolipoprotein E-deficient (ApoE−/−) mice with abscisic acid can ameliorate atherosclerosis by suppressing immune cell recruitment into the aortic root wall and upregulating aortic endothelial nitric oxide synthase expression [72]. They also suggested that, considering the findings of the former study by Magnone et al. [71], abscisic acid may be a part of a lesion-reducing mechanism [72]. Whatever the role of abscisic acid in the atherosclerotic plaque is, its function in inflammation (and presumably atherosclerosis) is mediated by LANCL2 [73]. The increased plasma LANCL2 levels of CVD patients found in our study, in combination with the observed increase of abscisic acid in plaques [71], further supports the proposed scheme.

Further proteins identified in this study and of special interest for further evaluation may be those that take part in cardiovascular system-related processes based mainly on animal model studies, but have not been studied in the human disease yet. DNA topoisomerase 2-beta (TOP2B) is such an example. In the study by Zhang et al. cardiomyocyte-specific deletion of Top2b protected mice from the development of progressive heart failure induced by doxorubicin [74]. Doxorubicin inhibits TOP2B, generating DNA double-strand breaks that are cytotoxic, induce pro-apoptotic DNA damage response and can eventually lead to cardiomyocyte cell death [74, 75]. Nevertheless, a functional connection of the observed increase in plasma TOP2B of CVD patients and the disease remains elusive.

Even though informative, our study also has limitations: the sample size is small especially considering the disease complexity, corresponding to a low statistical power. This largely stems from the fact that these samples were collected in the context of a prospective study where collection of tissue was also targeted to confirm disease pathology. To decrease, as possible, potential artifacts as a result of impact from this limitation, we applied stringent criteria for differential expression and targeted to verify findings based on dataset cross-checking (discovery versus test set; test set versus literature). In addition, one further shortcoming is that there is a significant mean age difference between cases and controls (65.8 versus 43.7 respectively). Nevertheless, following a closer data investigation (study expression levels of the specific proteins after omitting outliers per group and significance in the age difference; data not shown), the highlighted changes were not found to be age-associated.

## Conclusions

The proteomic analysis performed in this study, comparing plasma samples from CVD patients to control subjects, provides a comprehensive dataset to be used for further integrative studies in the future. The observed protein changes reflect known CVD-related processes such as changes in lipid uptake and inflammatory processes. Several novel findings are also highlighted forming the basis for hypotheses meriting further investigation including a potential pleiotropic role of LPR2 in CVD development but also links of SZT2 to hypertension regulation.

## Additional files

**Additional file 1.** Discovery set analysis results. List of peptides and proteins along with the detailed protein identification and relative quantification data per sample in the discovery set. The list of differentially expressed proteins is also provided.

**Additional file 2.** Pathway annotation results. Pathway (Cytoscape) analysis of the proteins identified in the discovery set. Pathways predicted based on proteins identified by at least two peptides are also shown.

**Additional file 3.** Test set analysis results. List of peptides and proteins along with the detailed protein identification and relative quantification data per sample in the test set. The detailed list of the differentially expressed proteins following the same expression trend (≥ twofold change) in both discovery and test sets is also provided.

**Additional file 4.** Ingenuity Pathway Analysis of the verified proteins. Lists of the canonical pathways and biological functions that resulted from the Ingenuity Pathway Analysis of the 39 verified proteins.

## Abbreviations

CVD: cardiovascular disease
LC–MS/MS: liquid chromatography–tandem mass spectrometry
PPD: plasma proteome database
GWAS: genome-wide association study
CE-MS: capillary electrophoresis-mass spectrometry
LDL: low-density lipoprotein
HDL: high-density lipoprotein
DTE: dithioerythritol
RT: room temperature
HCD: higher-energy collisional dissociation
FDR: false discovery rate
LXR: liver X receptor
RXR: retinoid X receptor
iNOS: inducible nitric oxide synthase
VLDL: very low-density lipoprotein
